# Global Neuromagnetic Cortical Fields Have Non-Zero Velocity

**DOI:** 10.1371/journal.pone.0148413

**Published:** 2016-03-08

**Authors:** David M. Alexander, Andrey R. Nikolaev, Peter Jurica, Mikhail Zvyagintsev, Klaus Mathiak, Cees van Leeuwen

**Affiliations:** 1 Brain and Cognition Research Unit, KU Leuven - University of Leuven, Leuven, Belgium; 2 RIKEN Brain Science Institute, Wako-shi, Japan; 3 Department of Psychiatry, Psychotherapy and Psychosomatics, Medical School, RWTH Aachen University, Aachen, Germany; 4 Kaiserslautern University of Technology, Kaiserslautern, Germany; University of British Columbia, CANADA

## Abstract

Globally coherent patterns of phase can be obscured by analysis techniques that aggregate brain activity measures across-trials, whether prior to source localization or for estimating inter-areal coherence. We analyzed, at single-trial level, whole head MEG recorded during an observer-triggered apparent motion task. Episodes of globally coherent activity occurred in the delta, theta, alpha and beta bands of the signal in the form of large-scale waves, which propagated with a variety of velocities. Their mean speed at each frequency band was proportional to temporal frequency, giving a range of 0.06 to 4.0 m/s, from delta to beta. The wave peaks moved over the entire measurement array, during both ongoing activity and task-relevant intervals; direction of motion was more predictable during the latter. A large proportion of the cortical signal, measurable at the scalp, exists as large-scale coherent motion. We argue that the distribution of observable phase velocities in MEG is dominated by spatial filtering considerations in combination with group velocity of cortical activity. Traveling waves may index processes involved in global coordination of cortical activity.

## Introduction

### Local modules and large-scale traveling waves

Current neuroscience tends to regard the cortex as a network of semi-autonomous regions [1]. Considerable progress has been made in determining the functions of individual regions at various scales, from cortical columns to whole Brodmann areas. Since these functions are hardly ever performed in isolation, fMRI (functional magnetic resonance imaging), EEG (electro-encephalogram), and MEG (magneto-encephalogram) studies typically observe activity distributed among multiple centers. Such observations have raised interest in how the centers are coordinated [2,3] and, in particular, the role of synchronous neural oscillations over multiple frequency bands therein [4–6]. The shift in focus from single to multiple interacting centers of activation has not changed the predominance, however, of the view that these activity centers are static.

What is becoming clear is that this view at best tells only part of the story. When summated over trials, brain activity may reveal localized activity maxima. Yet, especially outside the maximum regions, the activity also contains important within-trial structure. This structure often takes the form of traveling waves (TWs) [7].

A growing literature considers the functional significance of TWs at multiple spatial scales. These include the columnar scale [8,9] Brodmann area [10–13] and in large scale cortex [14–17]. How these different scales interact is an interesting question in itself, but in the present research we focus on the largest scale of TW dynamics. Herein we explore the hypothesis that the global phase trajectory of TWs captures important information about the coordination of functional brain activity.

Analysis of global TWs has had a long, if somewhat sparse and sporadic history [18–26]. Notably, as reviewed by Shevelev et al., 2000, there is a long tradition of research on the topic published in the Russian language. A possible reason for the paucity of present-day research into TWs is the ubiquity of fMRI as a measurement technique and its emphasis on difference imaging and functional localization. Unfortunately, measures of blood metabolism as used in fMRI have time constants that are too slow to detect rapid motion of cortical activation inherent to cortical TWs, unlike EEG and MEG.

Globally coherent TWs in the EEG/MEG may reflect mechanisms involved in coordinating fast processes such as global binding [22], communication across large cortical distances [28,29], sequencing of activity [13], controlled access to memory processes [30], gating of information flow [17] and generalization of cognitive representations [31]. How exactly these mechanisms perform this role remains an open frontier for research. We propose the view that traveling wave velocity (propagation speed and direction) is an important diagnostic in characterizing task conditions.

We may distinguish between the phase velocity and group velocity of signals in the EEG. Phase velocity is a property of individual waves. However, waves in the cortex travel in packets, and each individual wave in the group may have slightly differing characteristics e.g. wavelength. It is not presently possible to measure the individual phase velocities from the scalp, so here we report the group velocities. The group velocity can be understood as akin to the "centre of mass" of the wave packet, comprised of a number of waves, each with their own separate phase velocity [32].

### Traveling Wave Velocity

In EEG studies, global cortical waves arise at a variety of temporal frequencies, from the sub-delta through to gamma bands [14,15,33–35]. Massimini et al., 2004 analyzed slow waves, ~0.8 Hz, during sleep. They found these waves to have a modal velocity of 2 to 3 m/s. Patten et al., 2012 measured the speed of theta waves (4–8 Hz) along the anterior-posterior axis of the scalp; the average speed was about 4.0 m/s. In the same experiment, alpha-band waves (8–12 Hz) were measured with a mean velocity of 6.2 to 6.5 m/s. The alpha band provides the most commonly reported velocity estimate in the literature, with velocity ranges from 4.0 to 8.0 m/s [27,28,30,36,37]. Fellinger et al., 2012 report speeds for the lower (8–10 Hz) and upper (10–12 Hz) alpha bands—these were 6 and 8 m/s, respectively.

Overall there appears to be a tendency for speed to increase with temporal frequency. This observation is supported by Patten et al., 2012 and Fellinger et al., 2012, in which two temporal frequency-bands were measured; both reports show a commensurate increase in speed with temporal frequency. Indeed, we will observe that speed is proportional to wavelength at a fixed temporal frequency, and therefore as temporal frequency increases, so will speed if we focus on long wavelength waves.

### Phase velocity in the MEG using whole head sensor array

There have only been a few estimates of TW velocity in MEG cf. [22,28]. Yet, the MEG offers distinct advantages over EEG for observing wave phenomena. In MEG the magnetic field generated by the brain is directly measurable at each sensor site. In the EEG, the electric potential is measured across a channel comprised of two sites, as a difference in activity or phase between two sites. This complicates the interpretation of the EEG signal. Besides this, choice of reference also alters the filter characteristics of the EEG, both in the temporal frequency domain and spatial frequency domain [1]. MEG, being reference free, is not prone to these ambiguities. MEG is still subject to volume conduction, the blurring of signal by intervening tissues, but less so than EEG [38]. According to Burkitt et al., 2000, this makes velocity estimates from MEG recordings the more accurate ones. For these reasons we analyze MEG recordings.

Two previous reports on TW velocity in MEG exist in the literature [28,35]. One reports a mean velocity in the alpha-band of 5.1 m/s, though this estimate was fissure corrected, so the values measured at the scalp are lower [28]. The other study observed TWs in the gamma band that take 12.5 ms to traverse an array covering approximately half the scalp [35]; this amounts to an approximate speed of 10 m/s. Broadly speaking, both these results are consistent with the observation in EEG that speed tends to increase with temporal frequency. In the present research we estimate velocity of MEG TWs at temporal frequencies in the range 0.5 to 28 Hz.

### Traveling Wave Trajectories

An important component of wave velocity is its direction of motion. Wave trajectories vary with time, frequency and task [15,33,39]. However, much of the previous work on TWs has used a one-dimensional linear array of sensors to measure velocity [40]. Examples in the EEG literature include Burkitt et al., 2000; Fellinger et al., 2012; Freeman et al., 2003; Ito et al., 2005; Klimesch et al., 2007, and; Patten et al., 2012. This can lead to underestimation of the spatial frequency of the waves; when cutting diagonally across the axis of measurement they will appear to have longer wavelengths than is actually the case. This in turn will confound the velocity estimate. In addition, because linear arrays under-sample the spatial distribution of the waves, it is ambiguous whether progressive phase changes observed reflect a traveling wave or are generated by a small number of dipoles [28]. The under-sampling of the signal also means that the number of waves will be underestimated, particularly by ignoring waves moving orthogonal to the axis of the array. Notable exceptions to the use of linear arrays include Massimini et al., 2004 and Shevelev et al., 2000.

In the present work, we use whole head MEG arrays to characterize the *group velocity* of the waves; both their speed and direction. The TW measure that we use is an estimation of the peak value of the spatio-temporal power spectrum. This peak is found after normalizing the amplitude due to the temporal frequency alone. That is, for a given temporal frequency, we consider only the *phase* of the signal at that temporal frequency, discarding the amplitude, and then find the peak in the spatial frequency spectrum. In other words, we look for coherent spatial patterns in the temporal phase.

We can perform a simple test to confirm whether these waves exist in unfiltered data. The alpha band often contains clear peaks in the temporal frequency spectrum, even in short segments of MEG. For these epochs, if there is also a clear peak in the spatial frequency spectrum, then there is often a clear traveling wave in the raw, unfiltered MEG recordings. Our numerical methods enable the detection of TWs even where there is not a dominant peak in the temporal frequency spectrum. We can therefore consider how the value of the peak in spatial frequency spectrum of phase rises and falls over time and over a range of temporal frequencies.

At the peak spatial frequency we also consider the direction, wavenumber and velocity of the corresponding wave. We confine ourselves to phenomenological measures: the waves are characterized as a flow of phase across the scalp in the three dimensional coordinates of the measurement array. The reason for this simplification is our interest in defining relevant global states of the cortex as a single trajectory of activity. Given that our candidate property for global integration is the trajectory of the overall flow of phase, this property does not depend strongly on the underlying coordinate system used (i.e. scalp coordinates or cortical coordinates) nor on the smaller scale details of the underlying cortical topography (i.e the pattern of gyri and sulci). Our conclusions hold without an extra step of converting waves back to cortical coordinates and, as we shall argue, the measured waves are already filtered by the process of transmission through the scalp and therefore characteristic of only a subset of waves recordable at the surface of the cortex.

### Spatial frequency and standing waves

All the research reviewed herein is highly consistent in at least one regard. The waves measured at the outside of the skull are all of long wavelength, specifically, the range of phase in radians, across the scalp, is approximately 2π. In the discussion we cover in detail the implications of this issue, and its relationship to volume conduction. This subset of cortical waves with long wavelength dynamics may have functional advantages for integrating global cortical activity. This is because each unique phase corresponds to a contiguous region of the cortex and only the loci at equivalent points on the wave are bound at zero lag synchrony [31,41].

TWs of this global scale are pervasive in the cortex, and their detection is not strongly dependent on whether EEG, MEG, or ECoG (electro-corticogram) is used for measurement; trial-averaging, however, destroys information about TWs [7]. Therefore, here we analyze the property of phase velocity in single trials. We examine a wide range of temporal frequencies, from the slow wave to the beta band, in order to assess the relationship of frequency band to wave propagation velocity. We use a whole head array of sensors so that the trajectory and wavelength can be adequately characterized. We introduce to our analyses a novel method to distinguish between TWs and standing waves.

Contrasting with a traveling-wave perspective, methods for analysis of large-scale patterns in EEG and MEG generally assume standing wave-like phenomena. The default pattern is one in which the peaks arise at a particular location—described either as a maximum in activity or the site of an oscillatory process. For example, independent components analysis has been used to identify oscillatory patterns in the EEG, which can further be used to localize generators in the cortex [42]. By definition, assuming standing waves in the underlying model of cortical dynamics ignores the velocity of waves.

In fact, cortical waves can either be traveling or standing waves, and indeed different gradations in-between [28,43]. However, these gradations create an ambiguity in the relationship between wavelength and propagation speed. Standing waves arise out of interference between traveling waves, and therefore do not constitute a distinct, either/or phenomena [32,44]. As stated before, we only have access to the group velocity, not the phase velocity of individual waves. Speed is proportional to wavelength for pure traveling waves at a given temporal frequency. For standing waves, speed will be zero. Research into traveling waves in the cortex generally has not considered their relationship to standing waves. There has been no systematic quantification of wave propagation velocities that explicitly model gradations between traveling waves and standing waves. In the present research we build upon previous modeling of TWs in the cortex [7,45]; our model here includes two additional parameters: the weighting of a second, space-reversed wave and its phase offset. These additional parameters enable the characterization of a signal as a standing or pure traveling wave as well as all possible gradations in-between.

These gradations are important because if the wave model does not explicitly include them, the standing wave components can distort velocity estimates. In particular, treating standing wave components as if they were pure TWs can over-estimate the velocity. This is because the apparent velocity near the inflection point of a wave with a standing component provides an inflated estimate of velocity (approaching infinite velocity the closer to the wave is to a perfect standing wave). For the same reason, methods that estimate the velocity by integrating the wave motion over at least one cycle may also overestimate the velocity, since the estimate assumes a constant instantaneous velocity which is only accurate for a pure TW.

We found that TWs in MEG can be measured with a modal spatial period of ~30 cm, provided a sufficiently large measuring array is utilized. In estimating large scale TW phase velocity we find, consistent with our review of existing studies, that it scales linearly with temporal frequency. Velocity estimates for the wave events do not depend strongly upon selection criteria for these events. Also consistent with previous studies, the velocities of TW alter in distribution in a task-dependent and frequency specific manner. This occurs for wave speed but is even more striking for wave trajectories.

## Methods

### MEG Apparent Motion task

Twenty human subjects (age range 22–36 years, mean age 27.1; 12 female) engaged in an audio-visual perceptual task, while their brain activity was recorded via MEG. All subjects were right-handed, had no audiological abnormalities, and had normal or corrected-to-normal vision. Written informed consent was obtained from all subjects prior to participation in the study. The ethics committee of the University of Tübingen, Germany approved this study. The task required subjects to choose the direction of motion of an audio-visual apparent-motion stimulus. The visual stimulus, a white dot, was located on the horizontal meridian with the distance of 15° of visual angle at either side of the screen center. The apparent motion illusion was elicited by presenting the stimuli for 67 ms at one side, and then after 67 ms delay, for 67 ms at the opposite side. The auditory stimuli were white noise bursts, either to the left or right of the subject. The sounds were presented in such a way that they were spatially perceived at the position of the visual stimuli (+15° or -15°). The subjects reported a clear percept of motion of the stimulus. Subjects were instructed to trigger the stimuli by pressing a button with either the left or right index finger, with random choice for each trial. In some blocks the audio-visual stimulus moved from the side indicated by the subject and then to the other side. In some blocks of trials the direction of stimulus motion was randomized and not due to the subject’s choice. Further details of the experiment can be found elsewhere [46].

Neuromagnetic responses were recorded in a magnetically shielded booth using a 151-sensor whole-head gradiometer (CTF Systems Inc., Vancouver, Canada). Measurements were performed while subjects were seated. The MEG signals were sampled at 312.5 Hz. The time of the button press was designated as time zero in each trial.

Data were analyzed in each trial from −500 ms to +500 ms. In order to rule out possible confounds with the specifics of the task, and to show the generality of the results, some of the detailed analyses were carried out for both the motor portion of the task and the stimulus evoked portion.

#### Numerical Methods

We let *T*, *S*, *L*, *F* denote—respectively—the sample times, measurement sites (MEG sensors), trials and frequencies, as sequences of lengths *N*_*T*_, *N*_*S*_, *N*_*L*_, *N*_*F*_. Let *f*_*S*_ denote the sampling frequency of the digitized recording. The raw time-series signal is a 3-dimensional data set, *x*_*T×S×L*_. The Fourier components of the signal were estimated for a sequence of logarithmically-spaced center frequencies ranging from 0.5 to 28.0 Hz using 2 cycle Morlet wavelets. This range of frequencies was chosen as the maximum range that had good signal-to-noise ratio. Use of two cycles in the Morlet wavelets enables the phase and amplitude to be estimated from very short time windows, at the expense of frequency resolution [15,47].

The Fourier components are denoted *x*_*T×S×L×F*_. From the Fourier components we define:

Ψ=arg(X/‖X‖) (1)

which is the scalar-valued phase angle i.e. −*π* ≤ Ψ < π. In some contexts we refer to the complex-valued phase Φ = *e*^*i*Ψ^ = *X*/‖*X*‖. These quantities have the same indices *T*, *S*, *L*, *F*. For readability hereafter, some indices of *T*, *S*, *L* and *F* may be left implicit, depending on the context.

We define mean log power (MLP) as

$$\text{MLP}={\langle \text{log}({\left\|X\right\|}^{2})\rangle }_{S}$$ (2)

That is, the mean over the electrodes of the logarithm of the power. This quantity provides a useful comparison to the single-trial TW measures. Angle brackets indicate the mean over the index, in this case the mean over the sites, *S*.

### Traveling wave model

While traveling waves have been measured over a range of scales in the cortex, our present interest is in the global cortical field. In earlier research, the phase-gradient over the entire scalp has been measured by coherence analysis [48,49]. Coherence analysis is a measure of consistent phase (and amplitude) relationships across samples or trials. More generally, many EEG/MEG measures carry the assumption that measurements which are consistent across trials constitute the signal and measurements that are inconsistent constitute the noise [50]. Examples of such measures are ERPs and phase-locking factor and most source localization approaches. If this assumption is true, then these cross-trial aggregates dilute the noise and enhance the signal. Since this is an assumption of these measures, care must be taken in their interpretation in contexts where this assumption may not hold.

We have previously shown that models based on cross-trial averages do not capture as much variance within single trials as traveling wave models [7]. We argued that this poorer model performance is due to an incorrect assumption relating signal to cross-trial consistency. What counts as a typical wave-form cannot be characterized by averaging over trials since this destroys information about TWs [7]. Measures that are inherently cross-trial can only indicate the wave-velocity and wave-direction of the trial-averaged signal. However, traveling wave velocity varies in speed and direction from trial-to-trial, as we shall show. For these reasons we avoid such cross-trial measures in the present research.

We initially use *k*-means clustering to confirm the applicability of our wave model. The k-means was used to group similar phase patterns into clusters. That is, cluster membership is decided from phase information alone, without recourse to a model of large-scale dynamics. The input vector to the *k*-means was a *T* × *S* matrix, vectorized, and having length *N*_*S*_*N*_*cf*_. (*N*_*cf*_ is the number of samples in one cycle at the frequency of interest). Cluster membership was then used to compose *k* sub-averages of a subject’s brain signals, either by averaging the phase, or averaging the raw signal in units of Tesla, into the *k*-groups. This allowed a qualitative exploration of the data, but with fewer assumptions about the signal than the wave model. The *k*-means cluster provides a way to average over similar trials, without averaging over all trials and therefore assuming that cross-trial consistency equates with signal.

To analyze traveling waves we use Fourier related methods. The Fourier transform over time and multiple spatial dimensions is defined as

$$\underset{\text{n}}{\underset{︸}{\int_{-\infty }^{\infty }\cdots \int_{-\infty }^{\infty }}}f\left(t\right)e^{-2\pi it\cdot x}d^{n}t$$ (3)

for **t**, **x**∈R^n^. For the case of whole head MEG and EEG arrays, the application of this transform is not straightforward, for a number of reasons. The measurement arrays are (1) *not regular* lattices, and the measurement sites tile only (2) *part* of the (3) *surface* of an of an object in three spatial dimensions which is only by (4) *approximation* a (5) *spheroid*, on which the (6) *wavelength* can sometimes be greater than the size of the array. Even though methods exist to address each of these problems, for example, least-squares spectral analysis for problem (1), addressing them jointly is complicated. Fortunately, the following observation simplifies matters considerably.

We observed that much of the variance in single-trial phase can be captured by a traveling wave model with relatively simple mathematical form. In order to characterize typical wave-forms without resorting to taking a single average over all cases, *k*-means clustering has been used [16]. In the same spirit, principal components analysis has been used to decompose the spatial patterns of phase into basis functions [7,15]. Both these methods indicate the most common wave-form to be a linear gradient phase, Ψ (here scalar valued), described by

$$\Psi_{S}=\text{cos}(\phi (O[P_{S}]))$$ (4)

where *P*_*S*_ gives the coordinates of each measurement site projected onto the surface of a sphere, *O* rotates the origin of the spherical coordinates to the bullseye of the phase gradient, and the function *φ*() gives the polar angle (the latitude) in the spherical coordinate system. Waves travel outwards from the bullseye. If we refer to the bullseye where the waves radiate outwards as the north pole, then lines of equal phase travel outwards from the north-pole until they converge on the south-pole. The spacing of lines of equal phase are equidistant with the cosine of the latitude, φ, that is, equidistant along a line traveling from the north-pole, through the centre of the sphere, to the south-pole. This property of the waves makes for a useful simplification in their measurement.

The class of waves described by Eq 4 can be parameterized as a 3D vector within the Cartesian coordinates of the measurement array

$$\Phi_{S}=e^{i\beta \cdot P_{S}}$$ (5)

where *P* are the 3D Cartesian coordinates of the measurement sites, and form the basis to describe the waves. The *β* weights describe the wave vector in this Cartesian coordinate system. The weights play the role of rotating the model phase gradient (O[] in Eq 4), so that the gradient is aligned to the general direction of flow of phase over the scalp. The *β* weights also stretch the waves to allow for changes in wavelength over the scalp. The weights therefore play a role equivalent to Fourier components for spatial dimensions in Eq 3, for the case where **x**∈R^3^. For spatially unwrapped phases with scalar values [51], Eq 5 becomes

$$\Psi_{S}=\beta \cdot P_{S}$$ (6)

This phase gradient, Ψ_*S*_, is linear when expressed in Cartesian coordinates and its direction aligns with the north-pole, centre, south-pole line described for Eq 4.

We previously confirmed the explanatory power of the linear wave model using principal components analysis. In the present data, more than half of the variance in the phase gradient was explained by the linear gradients of scalar-valued phase (see Alexander et al., 2013). That is, the eigenvectors with the largest eigenvalues describe a pattern of phase whose wavenumber (spatial frequency) is approximately equal to one over the diameter of the measurement array. These large-scale waves therefore correspond to the dominant eigenmodes of the phase data, although their predominance in the signal is often overlooked, because it is washed out in averaging the data prior to or during analysis. Rather than treating these modes as an artefact of volume conduction, an artefact of spherical harmonics, or blurred signal requiring deblurring by source localization methods, we take the filtering by the skull and other tissues as an opportunity, enabling us to study the large-scale dynamics of the cortex. As stated in the introduction, our purpose is to characterize waves at the largest scale of cortex, in the context that waves occur at many different spatial scales of cortex. In other words, we are interested in characterizing the global spatial mode of the cortex, something which can be summarized as a single vector describing the flow of phase across the entire cortical sheet.

The weights *β* are parameterized by satisfying the expression

$$\begin{array}{c} \text{max}\\ \beta \in {\text{R}}^{3} \end{array}\left|{\langle \frac{\Phi_{j}}{e^{i\beta \cdot P_{j}}}\rangle }_{j}\right|$$ (7)

where *j* ∈ *S*. We find *β* by multi-grid search when calculating spatial frequency spectra for the TWs (see below). Since this method is computationally expensive we calculated the time by frequency plots by means of a numerically more efficient method. In this second method, we satisfy the following expression

$$\begin{array}{c} \text{min}\\ \beta \in {\text{R}}^{3} \end{array}{\langle \Psi_{j}-\beta \cdot P_{j}\rangle }_{j}$$ (8)

using ordinary least squares. This expression is directly analogous to Eq 7, swapping scalar-valued phase for the complex-valued phase.

To summarize, the model travelling waves are a linear gradient of phase over the surface of the volume defined by the measurement sites. Expanding Eq 6, this phase-gradient over the sites *S* is described by

$$\Psi_{\text{S }}=\beta_{AP}P_{AP}+\beta_{IS}P_{IS}+\beta_{LR}P_{LR}+\beta_{0}$$ (9)

where *β* are coefficients that describe the rotation and stretching of the phase gradient over the coordinate space of the sensors, *P* = (*P*_*AP*_, *P*_*IS*_, *P*_*LR*_). *AP* here refers to the anterior-posterior axis of the measurement array, *IS* to the inferior-superior axis, and *LR* to left-right axis.

The respective scalar components of the *β* trajectory are referred to as the anterior-posterior component, the inferior-superior component and the left-right component. These terms specify the *orientation* of the gradient. We refer to the *direction* of traveling waves using terms such as anterior-to-posterior trajectory, or a trajectory in the posterior-inferior-left direction. Positive values for each direction component indicate a wave going in the direction proscribed by the name e.g. positive anterior-posterior component indicates propagation in the anterior-to-posterior direction. Negative values indicate the direction is reversed; for example a posterior-to-anterior direction.

Relating these components back to Eq 3, the vector *β* is the spatial Fourier component with maximum power for a given temporal frequency. This is true for Eq 7, since we are finding, by brute force search, the spatial Fourier components that maximize the power. When there is a dominant peak in the spatial frequency spectrum, and such is the case for EEG and MEG (see results), then Eq 8 approximates the Fourier component of the peak in the spatial frequency spectrum. We have confirmed this by comparison of the estimates provided by these two methods (see S1 Fig). This means that the wave components produced by the two methods can be regarded as equivalent for present purposes, although the scalar case is more noisy due to errors introduced in the unwrapping procedure.

In order to compute the scalar valued phase, the measured phase was spatially unwrapped [51] to give *Ψ*_*S*_. We convert the phase into unwrapped phase by taking the discrete spatial derivative and then reintegrating spatially. The chief reason for this is that the spatially unwrapped phases allow the representation of a wave as a gradient over a field of scalars and thereby allow analysis by linear methods. Nearest neighbour edges on the measurement array *e*_*ij*_; *i*, *j*∈*S*, are determined by Delaunay triangulation [52]. For the phase at some *t*, *f*, and *q*, the phase difference on each edge is defined as *δΦ*_*ij*_ = arg(*Φ*_*i*_)−arg(*Φ*_*j*_)+2*kπ* with integer *k* such that *δΦ*_*ij*_∈[*−π*, *π*). We assign unwrapped phase values *Ψ*_*i*_, *i*∈*S*, using nearest neighbor phase relationships *Ψ*_*i*_ = *Ψ*_*j*_+*δΦ*_*ij*_. Edges are added sequentially, in ascending order of |*δΦ*_*ij*_|, to an initially edgeless graph with sites as vertices. If |*δΦ*_*ij*_|<<*π* is not everywhere true, unwrapping errors can arise, where *Ψ*_*i*_−*Ψ*_*j*_ = *δΦ*_*ij*_+2*kπ* for some integer *k*≠0. However, the number of such errors is small (compared to the number of *e*_*ij*_) in data where phase varies smoothly i.e. during a coherent TW. Unwrapping errors may either reflect legitimate discontinuities in the phase or measurement noise (Spagnolini, 1995). Adding edges in ascending order of |*δΦ*_*ij*_| places unwrapping errors in regions of highest |*δΦ*_*ij*_|, i.e. at edges that are either legitimate discontinuities or due to high measurement noise.

### Wave map, wavenumber and velocity

The wave map constitutes the traveling wave model after it has been projected back onto the coordinates of the measurement array i.e. the model represented in the same units as the measured phase. The direction and wavelength of the phase gradient can be described by a single three dimensional vector, namely *β* = (*β*_*AP*_, *β*_*IS*_, *β*_*LR*_). *β* is therefore a shorthand for describing the wave map (but not to be confused with Beta temporal frequency). The wave map is a map in the sense that it conveys the topography of phase. Due to the equivalence of expressions 7 and 8 in estimating *β*s, we confine the rest of the exposition to describing the complex-valued case. The *wave map*, Θ_*S*,*q*_, computed from the measured phase over all sites *S* at trial *q* is then:

$$\Theta_{S,q}=e^{i(\beta \cdot P)}$$ (10)

This formulation differs slightly from Alexander et al. (2006b, 2008, 2009), where the wave map (or phase gradient) was expressed in the metric of a linear gradient of relative phases, i.e. without converting to a complex number. The relationship between the two can be seen by considering the scalar-valued equivalent of the wave map:

$${Ο}_{S,q}=\beta \cdot P$$ (11)

The spatial frequency, or *wavenumber*, of the wave map is given by

$$\xi =\sqrt{\beta_{AP}^{2}+\beta_{IS}^{2}+\beta_{LR}^{2}}$$ (12)

The spatial frequency is given as cycles/meter in sensor coordinates rather than in terms distance traveled around the curvature of the measurement array or cortical coordinates (see Discussion). We compute the spatial frequency spectrum by finding the *β* that best satisfies Eq 12 for each value of *ξ* in the range *ξ* <10 cycles/m (minimum wavelength 2.5 cm). The spatial frequency power is expressed in normalized units (zero to unity), as the fit of the wave map to the phase according to Eq 12. Spatial frequency was also calculated over all possible array sizes, from 7 sensors to 151, for each possible center location of the sub-array. This was done to check the effects of array size on wavenumber estimates.

We calculate the velocity of the wave over all sites and over a window of one temporal cycle at the frequency of interest (e.g. 100 ms for 10 Hz). This time window of width 1/*f*, centered at sample *t*, is defined as follows. Let *C*_*f*_ denote a sequence of time offsets at frequency *f*: $C_{f}=(c_{1},c_{2},\ldots ,c_{N_{c}})$ where {c1,c2,…,cNc}=(−12f,12f)∩(ℤ/fs), where ℤ denotes the integers. Thus $N_{C_{f}}\approx f_{S}/f$. An input matrix of phases of dimension $N_{s}\times N_{C_{f}}$ was used in the estimation of wave velocity. $N_{C_{f}}$ was also used in the definition of the input vector into the *k*-means analysis, described earlier. Since the wave map is defined for a single sample, we first extended the wave map over a window of one cycle to allow comparison with the normalized ER:

$$\theta_{S\times C_{f},q}=∠\Theta_{S,q}+2\pi fc;c\in C_{f}$$ (13)

$\theta_{S\times C_{f},q}$ corresponds to the (scalar-valued) phase of the model traveling wave. ∠ denotes the argument (angle) of a complex number.

By definition, for traveling waves of a specific spatial frequency, speed of propagation is proportional to temporal frequency. Now, we generalize the wave model to allow for a range of velocities at a given temporal frequency and wavelength. We allow for variation in wave speed by modeling a range of behaviors, from standing waves to traveling waves. This range can be expressed as the normalized speed: the number of spatial cycles traveled per temporal cycle, for a given temporal frequency. We elected to represent wave speed in a manner that was not dependent on temporal frequency, since the dominant spatial frequency of the waves measurable outside the scalp does not greatly change with temporal frequency (see Results). This choice has the advantage that different temporal frequencies have comparable units of speed. Normalized speed is therefore defined so that it varies from zero in the case of pure standing waves to unity in the case of pure traveling waves.

We treat Eq 13 as the positive component of the generalized wave, and add to it a space-reversed version [53] of the same wave:

$$\omega_{S\times C_{f},q}=\alpha_{p}\text{cos}(\theta_{S\times C_{f},q}+\theta_{p})+\alpha_{n}\text{cos}(\theta_{(N_{S}-S)\times C_{f},q}+\theta_{n})$$ (14)

where *α*_*p*_ and *α*_*n*_ are positive scalars giving the weightings of the positive and negative going portions of the generalized wave, and *θ*_*p*_, *θ*_*n*_ are corresponding phase offsets. The model wave is fitted to one cycle of the real part of the complex-valued phase, $ℜ(\Phi_{S\times C_{f}})$, using ordinary least squares. When *α*_*p*_ = *α*_*n*_ the model wave is a standing wave. When *α*_*n*_ = 0 the model wave is a pure traveling wave. The normalized speed of the wave is the minimum instantaneous velocity over *C*_*f*_ (i.e. the instantaneous velocity at the peak), expressed in units of spatial periods traversed per temporal period.

$$\left|v\right|=\frac{\alpha_{p}-\alpha_{n}}{\alpha_{p}+\alpha_{n}}$$ (15)

This definition avoids spuriously fast speed estimates. In the case of a pure traveling wave, all points on the wave move at a constant speed, according to Eq 15, at a normalized speed of unity. However, in other cases, estimating the speed at points other than the peak of the wave will artificially inflate the estimate, particularly in those waves that have a large standing component. For example, in the case of a wave which is almost a perfect standing wave (*α*_*n*_ is equal to *α*_*p*_), the lowest measurable “instantaneous” velocity is close to zero. This occurs at the peak of the wave and gives the desired value for speed. Our method of estimating normalized speed was therefore conservative in speed estimates because it is more accurate than methods which do not utilize the whole cycle, but instead estimate the phase velocity from a limited number of time-points (c.f. [9,13,14,30,36,49]

The fit between the wave map and the measured phase, hereafter referred to as the *wave activity*, is denoted Ω_*t*,*f*_. The wave activity is calculated here as:

$$\Omega_{t,f}=\rho (\omega_{S\times C_{f},q},ℜ(\Phi_{S\times C_{f},q}))$$ (16)

where *ρ* denotes the correlation function for two vectors. Each *ω* and ℜ(Φ) are vectorized and have length *N*_*S*_*N*_*cf*_. The quantity Ω_*t*,*f*_ is therefore the correlation between the empirical phase and the standing/traveling wave model. Since the α and *β* weights in the model are analogous to Fourier components, the fit is a (normalized) measure of power. Hence wave *activity*. This definition of wave activity differs from the one in Alexander et al., 2013, since the latter did not account for the speed of the wave and was computed for a single sample, rather than over one cycle. The present definition yields event-related features from the signal that are qualitatively similar in most respects to the previous definition of wave activity i.e peaks and troughs in wave activity occur at the same latencies and frequencies for most events.

### Permutation methods to test over-fitting and family-wise error

In calculation of wave activity, the wave model is estimated from the empirical phase, prior to calculating the fit of the model back to the empirical phase. Thus the final fit involves a circularity in the procedure. As a simple check that the wave activity was not over-fitting the data, we re-analyzed one subject. In this extra analysis we randomized the phase at each site and sample, in order to assess the correlations expected from the fitting procedure alone.

As stated, the fit of the model waves to the empirical phase were assessed via correlation. To gauge the event-related structure of these correlations, and associated Fourier components, we tested whether values differed from baseline levels. The baseline was calculated at each frequency over the time-range -500 ms to -250 ms. Statistical significance for each (*t*, *f*) ∈ *T* ×*F* was assessed using a Wilcoxon signed-rank test. Due to the large number of statistical tests that this entails (*N*_*T*_ ×*N*_*F*_ for each measure, for each subject/condition), the overall statistical significance for each time by frequency matrix was assessed by analysis of the spatial clustering of contiguous significant regions in the time/frequency matrix [33,54,55,56]. A region of significance was only included if the probability of generating a cluster of that size by chance (randomly permutating the times) was less than the significance level. This effectively reduces the number of tests of significance to one test per plot. The statistical differences from baseline were not central to the arguments presented in this paper, except to illustrate the event-related nature of the signals, and therefore their likely functional relevance.

## Results

### Heterogeneity of cortical dynamics

The present study analyzes the class of waves which are characterized by a linear trajectory in the Cartesian coordinates of the sensor space. These waves account for a large proportion of variance in EEG and MEG signals [7,15]. Other classes of waves were, at times, observed in the raw and sub-averaged data, including spiral waves [10,16]. TWs in the MEG data are episodic in nature, like the EEG, ECoG and LFP (local field potential) [7,29,57]. Inspection of single-trial time by frequency plots reveals that orderly waves are usually detected at only one frequency at a time in EEG and MEG data, but there can be several episodes per second across the frequency range. We include some typical single-trial plots in S1 Fig.

**Fig 1:**
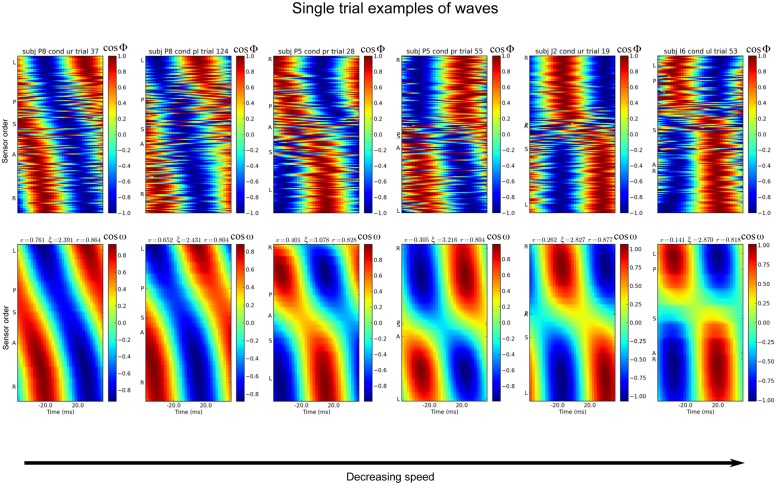
Examples of single trials with good fit to the wave model. The top row shows the cosine of the phase, at 9.2 Hz, across all sensors. Examples are taken from a variety of subjects and experimental trials. The times shown on the x-axis are relative to the center time of one cycle of the wave. The bottom row shows the model wave that was fit to the data. *v* is the normalized velocity of the wave, *ξ* is the wavenumber (cycles/m) and *r* is the fit of the model wave to the data. On the y-axes, some sensors are labeled to indicate the approximate spatial ordering of the wave: ‘A’ is the most-anterior sensor, ‘P’ posterior, ‘I’ inferior, ‘S’ superior, ‘L’ left, and ‘R’ right. The sensors are ordered along the y-axis by values of the wave map calculated from the phases of the center time-sample.

Some examples of global MEG waves are shown in Fig 1. The estimated direction of the wave, according to the wave map, is used to order the sensors along the y-axis of the plot (see methods). This ordering enables spatio-temporally smooth waves to become visually salient in the data, either as a diagonal stripe in the case of traveling waves or as a vertical, checkerboard pattern in the case of standing waves. When neither such pattern appears in the plot, no wave with these characteristics is present in the data. The figure shows some examples of both standing and traveling waves in the alpha band, as the cosine of the phase at 9.2 Hz and as the model fit, ordered by the velocity of the waves (left-most panels to right-most). An example traveling wave is shown in S1 Movie, and more examples can be found in [7].

In terms of neural mechanisms, the static maxima and minima in field strength seen for the standing waves (right-most panels) can be interpreted as being due to a localized, stationary source of brain activity [42]. It is difficult to interpret traveling waves this way, since the maxima and minima in field strength move over large distances (Fig 1, left-most panel).

**Fig 2:**
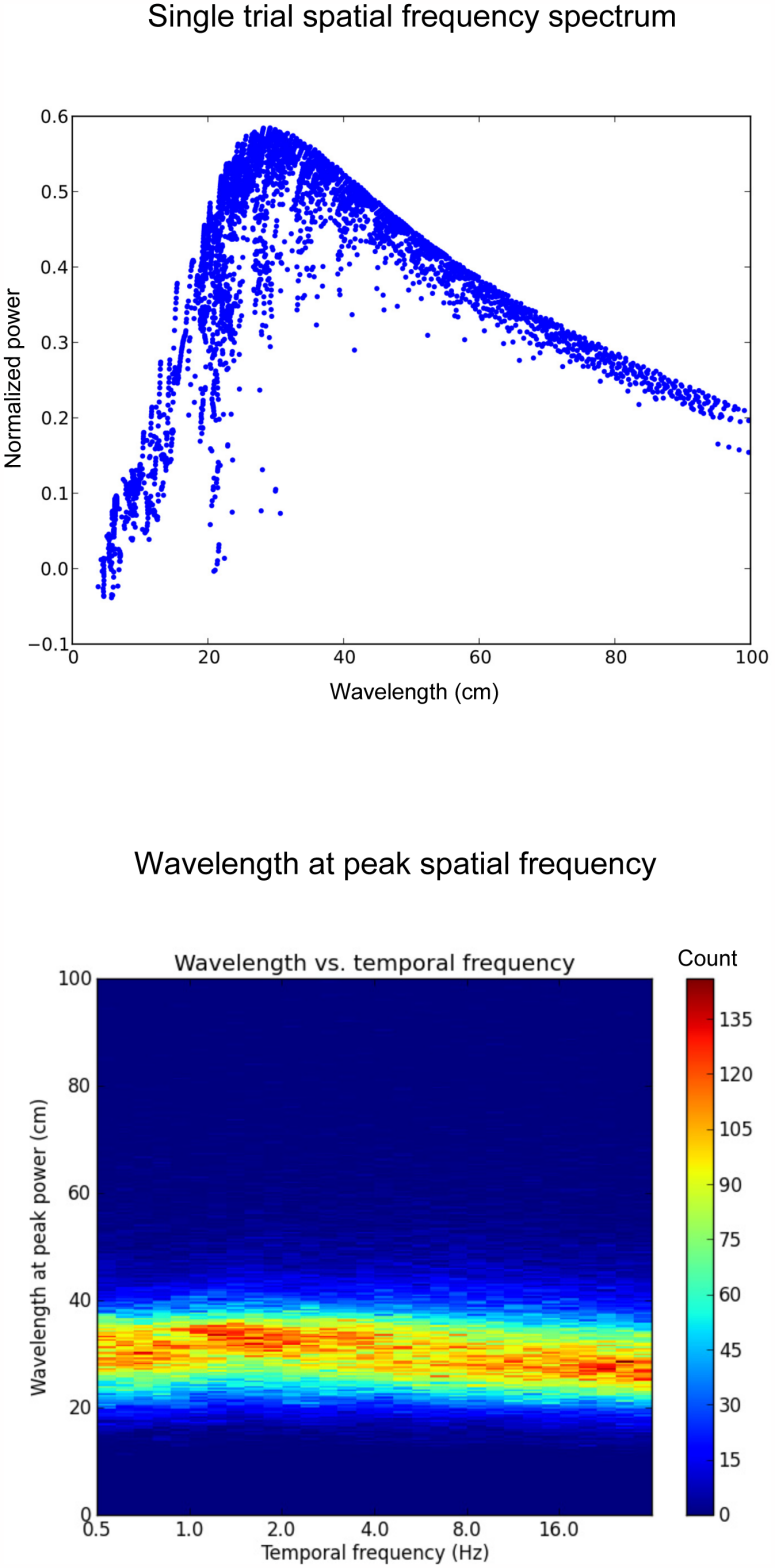
Typical wavelength spectrum and distribution of peak wavelengths over all samples. The top panel shows the spatial frequency spectrum for a single sample of one subject’s MEG. The peak in the wavelength spectrum in this case was at 27cm. The bottom panel shows the distribution of peak spatial frequencies over the entire population of samples. The bulk of waves have wavelength between 25 and 35cm, with delta band (1 to 4Hz) having slightly higher wavelengths compared to beta band (16 to 28 Hz).

Consistent with previous reports for the EEG and MEG [7,15,16,35], the present waves were typically of long spatial wavelengths, regardless of temporal frequency. This is illustrated in Fig 2, which shows the spatial frequency spectrum for a typical sample. Fig 2 also shows the peak in the spatial spectrum measured across the population of trials. The means of the spectral peaks distribution were between 25 and 35cm, with slightly longer wavelengths in the delta band (peak at 34 cm) and shorter ones in the beta band (peak at 30 cm).

The spatial period (wavelength) of the waves was therefore slightly larger than the diameter of the head, so only one cycle of phase was present across the global cortex. It is known that the skull and other tissues have the effect of a low pass spatial filter [1,23,38]. It seems unlikely that these non-cortical tissues generate apparent spatial frequency power where none exist, however (see discussion). The measured wavelengths were not strongly dependent on the size of the measurement array, for arrays of diameter 10cm or more (see S2 Fig). Consistent with Nunez's [54] analysis of EEG coherence, by varying the measurement array size we find a peak spatial frequency at < 5cm and another at > 20cm (see his Fig 9). Both classes of longer wavelength coherences have a genuine cortical origin, according to Nunez et al., 1997. This means the measured long wavelength dynamics are not an artefact of the shape of the skull i.e. of a limited number of sources measured from opposite sides of a spheroid c.f. [58]. We therefore conclude that the detected spatial frequencies, using whole-head arrays, reflect real cortical processes, with spatial frequencies smaller than 15 cm being attenuated by the intervening tissues. Since detection of these long wavelength phenomena are limited by the sensor array size, whole head MEG and EEG is suited to characterizing the long wavelengths of interest here. When very small measurement arrays were used, we observed a peak in wavelength at a smaller spatial scale (see S2 Fig), however, these waves are more ideally detected by high resolution techniques and ECoG [32,44].

We previously argued that the topography of ERs over the head is largely an artifact of averaging over individual traveling wave events during the process of data analysis [7]. A corollary of this claim is that the trial-averaged signal will not necessarily reflect the spatio-temporal structure of the underlying signal. By way of analogy, Moiré patterns do not show the same spatio-temporal features as the component images used to construct them. We explored the effects of averaging by using the *k*-means clustering. The clustering was used to decompose the signal into sub-averages based on their trial-wise similarity. The input vectors to the *k*-means were comprised of the phase of the signal (see methods): no information about traveling waves was provided.

**Fig 3:**
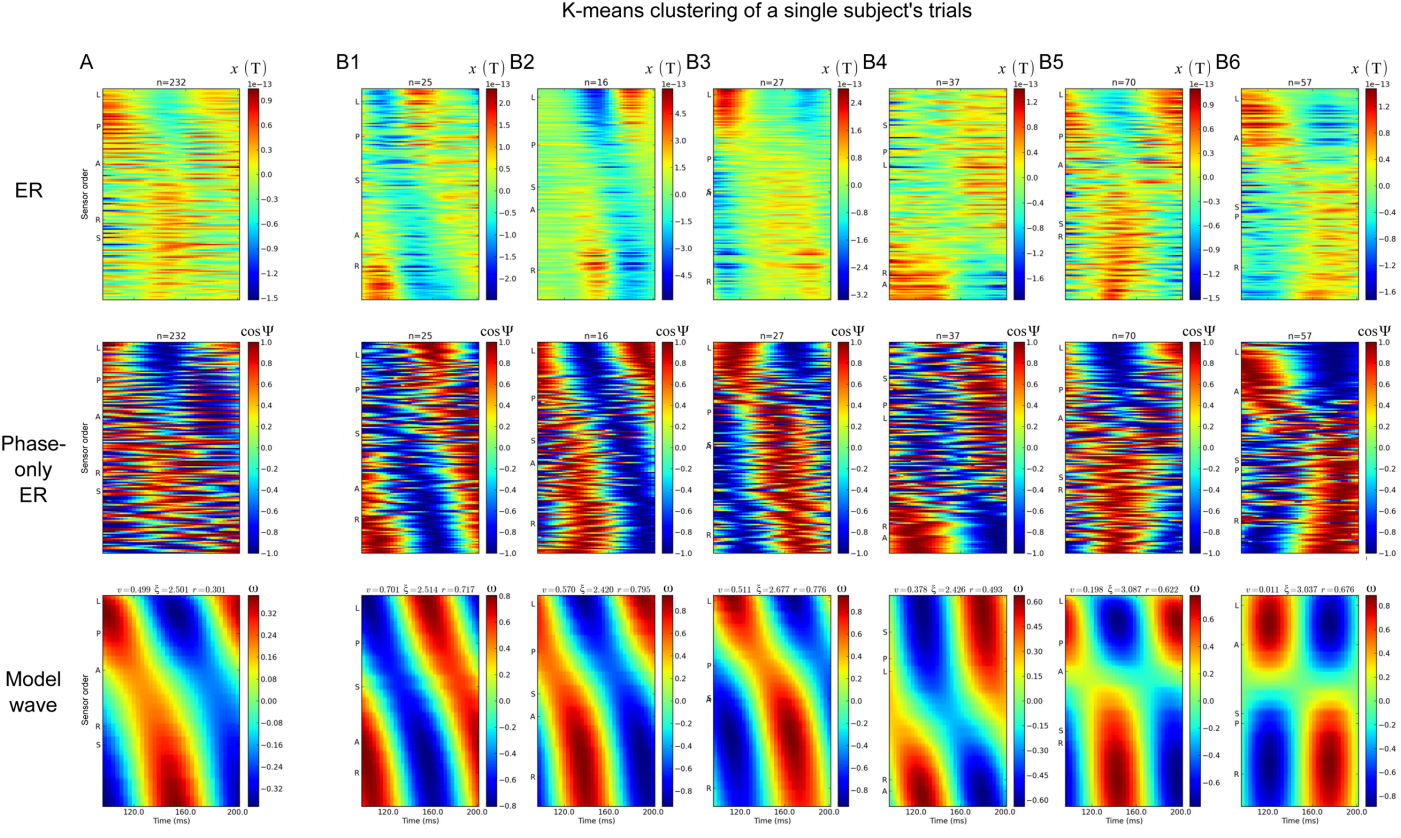
Clustering trials by phase values reveals a wide range of dynamics in sub-averages. These data are taken from subject ‘P8’, conditions predictable-left and unpredictable-left. The data are shown for one temporal cycle at 9.2Hz i.e. 108ms. Time at the center of the temporal window was 150ms post-button press. The trials were clustered using *k*-means (*k* = 6), with input vector being the phase at over the cycle centred at 150ms and over all sensors. A. The trial-averaged data from all the trials. B. The sub-averaged data from each of the six clusters. The top row shows the raw MEG signal, in units of Tesla. The number of trials in each average is indicated by *n*. The second row shows the cosine of the trial-averaged phase. The third row shows the model wave estimated from the trial-averaged phase. The sensor labeling on the y-axis is the same as for Fig 1.

As a benchmark for the clustering results we considered the fit of the TW model to the trial averaged ER, shown for one subject in Fig 3A. The spatial pattern shows the characteristic dipole pattern over the visual cortex (here at 150ms post-button-press). The model wave was estimated from the trial-averaged phase at 9.2Hz. It did not fit the data very well (*r* = 0.301). In other words, the trial-averaged signal did not look like a traveling or standing wave. If trial-averaging improves signal to noise, then we would expect model fits to improve with such averaging. This was not the case for the present wave models, indicating that trial-averaging of time-series does not improve signal to noise for this family of waves.

By contrast, a rich variety in dynamics was revealed by clustering the trials according to spatio-temporal patterns in phase. As example, the *k*-means clustering of data from the same subject is given in Fig 3B. Out of six clusters, three showed traveling waves moving from the left to the right side of the head—shown in Fig 3B1–3B3. These three sub-averages were offset in phase from each other, accounting for the lack of the appearance of a left-to-right traveling wave in the trial-averaged data despite its prominence in these sub-averages. This lack is due to destructive interference in the cross-trial average. Two of the sub-averages showed clear standing wave patterns (3B6, 3B7). All of these patterns were apparent in both the (cosine of) sub-averages phase (middle row) and sub-averages of the raw time-series (top row). The sub-averaged time-series indicate that the patterns were not a simple artefact of phase estimation, because the TWs can be seen in averages of the raw signal. The fits to the wave model were good (*r*>0.6) for five of the clusters, but not for cluster four.

These data particularly illustrate our point regarding heterogeneity of the measured dynamics. However, the same general observations were true of all subjects: sub-averaging the MEG data by the spatio-temporal patterns of phase revealed a broad variety of spatio-temporal patterns that were not visible in the trial-averaged signal. Next, we turn to an analysis of the range of velocities seen at the individual-trial level.

### The distribution of wave speeds and directions

**Fig 4:**
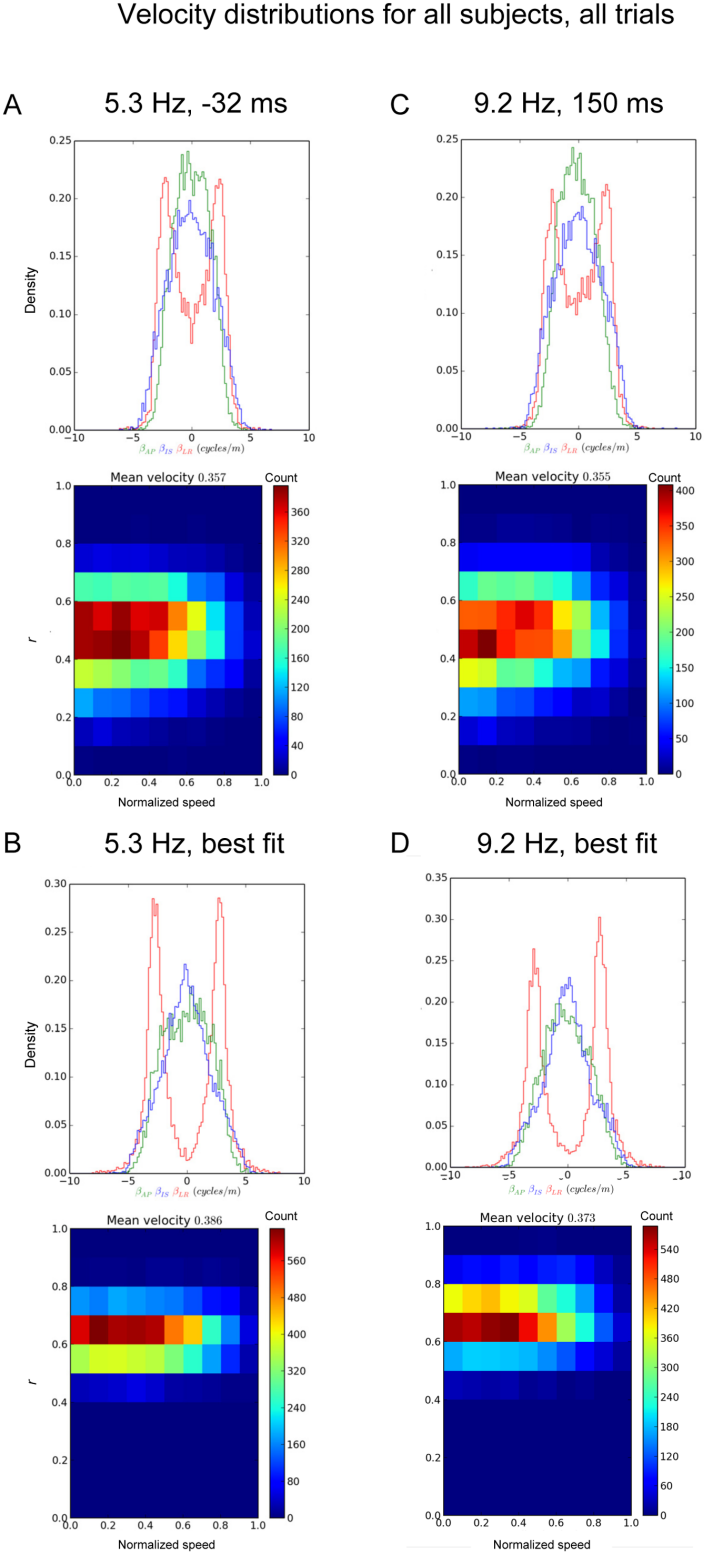
Distribution of wave velocities. A: Distribution of velocities at 5.3Hz, collated over all subjects and conditions at -32ms. Upper figure indicates the distribution of trajectories. Lower figure shows the speed of the waves. B: Same as A, except the best fitting wave from each trial is included, rather than the fit at a specific time. C: Distribution of velocities at 9.2Hz, 150ms, collated over all subjects and conditions. Upper figure shows the distribution of trajectories. Lower figure shows the speed of the waves. D. Same at C, except the best fitting wave from each trial is included, rather than the fit at a specific time.

We estimated the normalized speed within the trial at latencies coinciding with motor, visual, and auditory dipoles, at the characteristic temporal frequency of these events [7]. The distribution of normalized speeds at -32ms, 5.3 Hz is shown in Fig 4A, corresponding to the motor dipole event. The distribution is peaked at just over 0.35 but is rather uniform; wave speeds from zero and 0.8 can readily be found. Since events with good fits (e.g. *r* > 0.6) to the wave model were episodic in nature, the events with good fits form a small tail in the total distribution of fit values. This can be seen from the range of values for *r*, shown on the y-axis in Fig 4A, lower panel.

**Fig 5:**
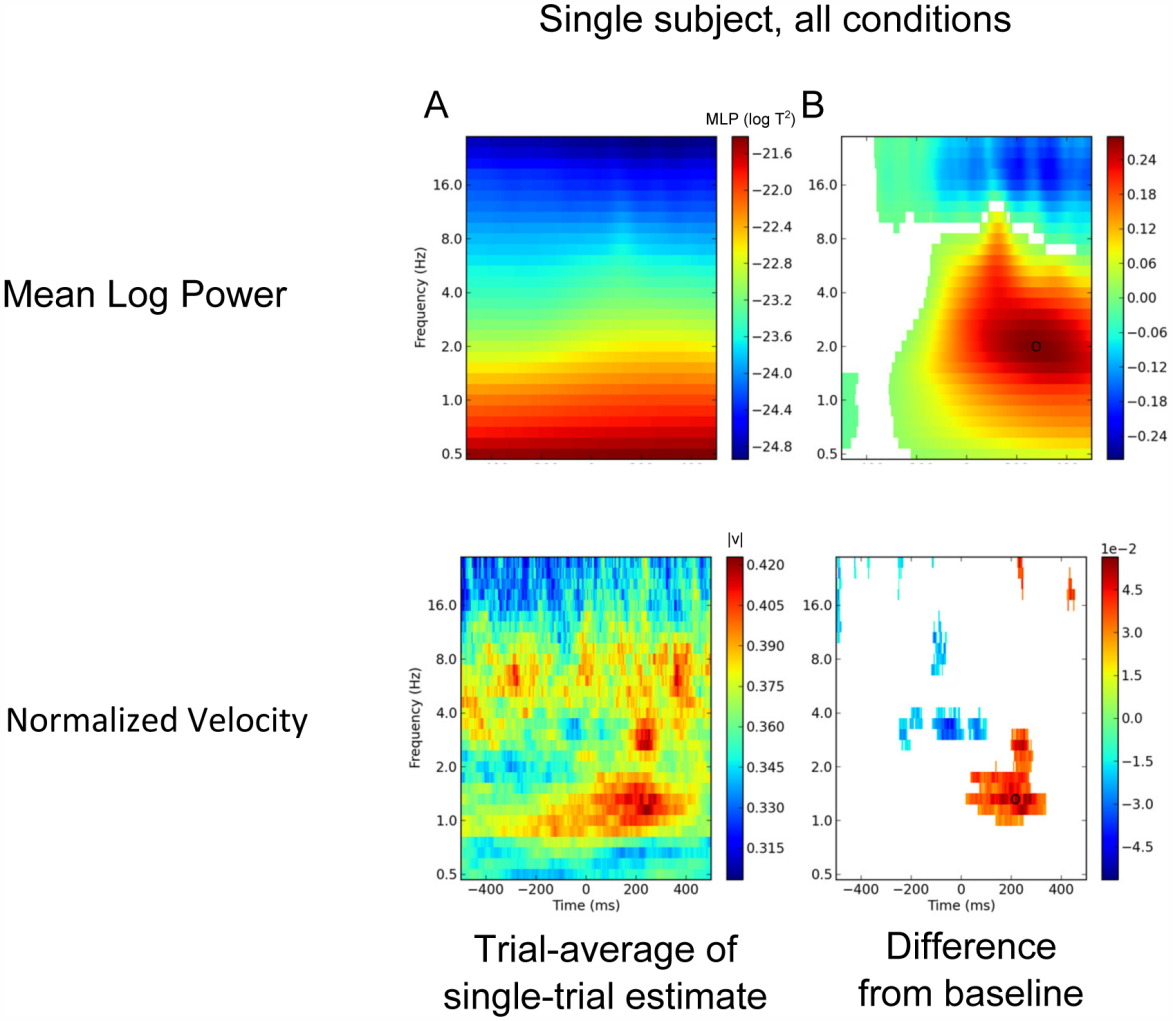
Single subject example of time by frequency statistics (subject H8). A. Mean log power and normalized velocity are averaged over all trials and all conditions. B. Regions that are significantly different (p<0.05, Wilcoxon signed-rank test) from the baseline level are shown in colour (*n*_trials_ = 475).

**Fig 6:**
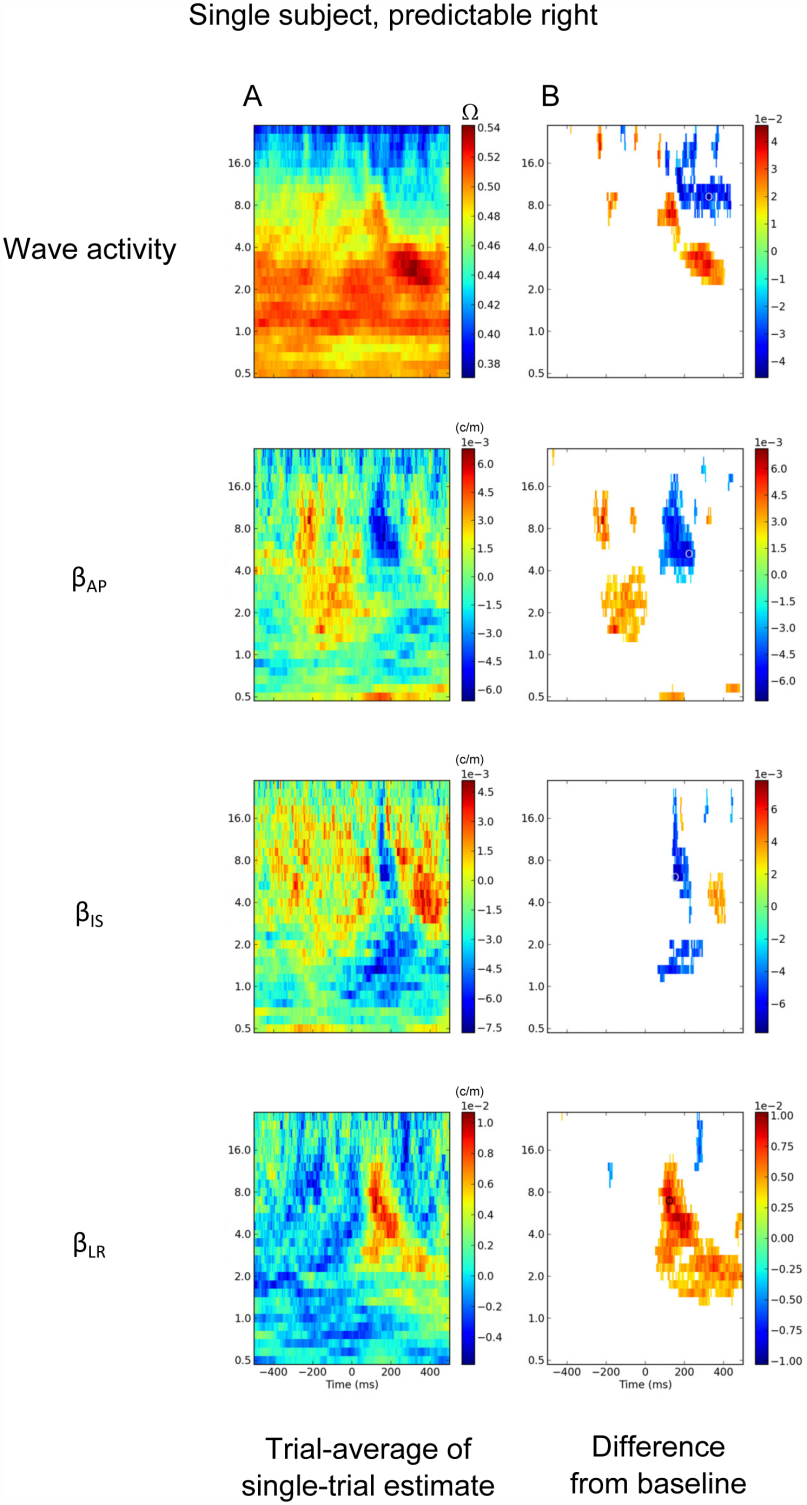
Single subject example of time by frequency statistics (subject O9). A. Wave activity, β_AP_, β_IS_ and β_LR_ are calculated at the single trial level, then averaged over trials. B. Regions significantly different (p<0.05, Wilcoxon, n_trials_ = 433) from the baseline levels for (A) are shown as non-white.

In order to assess the velocity measurements expected from the fitting procedure alone, we repeated our analysis of the single subjects’ results shown in Figs 5 and 6. S1 Fig shows the distribution of velocity statistics for a single trial when the phases are randomized. A lack of significant event-related regions, and the general noisy appearance of the plots compared to the non-randomized data, both indicate the results are not due to the fitting procedure.

We wanted to rule out the possibility that the traveling wave portion of the distribution was simply due to epochs with poor fits, i.e. modeling of noise. We used an alternative selection criterion for the analysis such that, for a given temporal frequency, the best fitting sample to the wave model during a trial was included, rather than the fit at a specific latency. This improved the average fit of the distribution, as expected, but did not noticeably change the distribution of normalized speeds (Fig 4, lower). We repeated this procedure over all frequencies, and the distribution of normalized speeds was quantitatively similar regardless of temporal frequency (0.38 in delta and theta bands, 0.37 in alpha and beta). The mean (non-normalized) speeds over the range of temporal frequencies equate to a range of 0.06 to 4.0 m/s for 0.5 to 28 Hz.

The waves in this analysis of MEG data also show wide variation in the values of the direction components; waves propagate over a range of possible directions. However, for the left-right component, wave events with a near-zero component tend to be less frequent than ones with a large magnitude. This is seen in Fig 4, in which *β*_*LR*_ has a bimodal distribution in each of the plots and, in head coordinates, where the scatter of wave directions shows a region of lower density along the mid-sagittal plane. This variation in trajectories can also be seen in S2 Movie, which shows all the wave direction vectors for all subjects at 1.3 Hz. The wave direction vectors scatter outwards from the two hemispheres of the cortex. It should be noted that the wave direction vectors are simply a shorthand for describing the flow of phase measurable over the surface of the scalp. These observations did not change by including only those events that best fit to the wave model within each trial, as seen in the lower half of Fig 4 in which the distributions of wave direction components are similar to the upper half, except the bimodal distribution of *β*_*LR*_ was more pronounced. This implies that some of the *β*_*LR*_ values near zero, seen in the upper half of Fig 4, arise because of poor fits to the wave model.

S1 Fig shows similar information to Fig 4, but across all frequencies and for a single trial. The regions inside the white lines have a fit of 0.7 or greater; outside the white lines the fit is poor. For each of the wave statistics of trajectory and speed, these single trial plots illustrate qualitatively how the distributions shown in Fig 4 are organized in time and frequency space at the single trial level, and how the distributions do not depend strongly on event selection criteria. This information about event selection criteria is also conveyed by Fig 4. The distributions found for the latency and characteristic frequency of the visual dipole (150ms, 9.2Hz) are broadly consistent with the results for the motor dipole, as were the results for the auditory dipole. Indeed all times and frequencies that we analyzed show related distributions of trajectories and speeds. This does not mean that the wave statistics did not vary in an event-related fashion, as we shall see in the next section.

### Event-related changes in velocity

Event-related changes in normalized speed are clearly in evidence in the single subject of Fig 5 as well as in the grand-average plot for speed in Fig 6: an increase in wave velocity in the delta band, 0–200 ms post-button-press. The peak normalized speed in Fig 5 has a mean of 0.38 at ~50 ms, 1.5 Hz. Note that the peak of this increase in normalized speed is earlier in latency than the (peak) event-related increases in MLP and wave activity, but its exact meaning is unclear at this point.

There were also event-related changes in the anterior-posterior component of the waves. These changes occurred in the region of 176 ms, 7.0 Hz when the waves, in deviation from their normal range of behavior, started to propagate predominantly in the posterior-to-anterior direction. See S1 Movie for a typical wave at this time and frequency. In this example, the wave event included a strong superior-to-inferior trajectory.

Fig 6 shows the average *β*_*AP*_ measure for a single subject, in the predictable right condition; that is subject selection of ‘rightward’ during a predictable stimulus block. The tendency for waves to have a posterior-to-anterior direction at around 176 ms, ~7.0 Hz is clearly seen, and this deflection from the baseline trajectory (Fig 6B), although it extends from theta to well into the beta range, was centered in the alpha band. For this reason, we consider this wave event to be in the alpha band. The poor frequency resolution resulting from the need to analyze short-lasting phase events means the exact frequency cannot be determined. At approximately the same time and frequency there were also changes in the other components: waves showed an increased tendency to propagate in the superior-to-inferior and the left-to-right direction (Fig 6B). The combined effects of these changes are shown in S3 Movie. For most time samples at 7.0 Hz, there is a broad scatter of wave trajectories across trials. However, at around 176 ms, the trajectories become more biased toward the anterior-inferior-right directions.

These event-related results are included here to illustrate that the measured velocities are functionally meaningful, and qualitatively similar to those event-related changes reported for Oddball tasks [15,33]. The task-related changes in wave velocities are analyzed in more detail in Alexander et al (ms. in prep.), along with their relationship to known dipole events, and cross-subject analyses. The single subject results reported here are typical.

Overall, wave events have broad distributions of both trajectories and speeds. The waves are ubiquitous, with clear episodes across the frequency range, several times a second. Waves with these properties have a clear event-related structure, pointing to their potential functional significance. The waves are long wavelength, at around 30cm, and speed increases linearly with temporal frequency. Thus we are able to confirm the pattern predicted from previous studies, over a wide range of temporal frequencies using uniform methods.

## Discussion

Patterns of whole-brain activity are increasingly analysed in terms of interactions between static, localized centers of activity. Such analyses reveal important facts about functional localization in the cortex, but may miss how activity on the cortical surface is distributed *in space and time* [28]. We analyzed traveling waves (TWs) in the MEG, elicited during a task where the subjects initiated an audio-visual apparent motion stimulus. We characterized linearly propagating waves in terms of resemblance to standing waves vs. TWs. We looked at the velocity of TWs over the entire range of temporal frequencies where there was good signal-to-noise ratio in the MEG signal.

### Large scale coherent waves

Much of brain research is focused on localization of function in networks of interacting, semi-autonomous modules. The present research, however, considers the globally coherent component of the cortical signal. This component explains a large proportion of the variance in phase [7,15]. The finding that the cortical signal is dominated by long wavelength components is consistent with the spatial spectra of cortical signal having the form $\frac{1}{f^{m}}$ [11]. The measured spectrum in the MEG is peaked at about 30cm, and we assume this is in part or in whole due to the skull and other tissues acting as a low-pass spatial filter. Our previous research has shown that MEG and EEG data, in general, have these approximate spectral characteristics [7,15,33]. Existing techniques for source localization involve removing this long range component of the signal, thereby (relatively) amplifying the residual high spatial frequency components. Here we take the spatial filtering effects of the extra-cortical tissue as an opportunity to analyze the long-range components of the signal.

### Velocity over the frequency range

Our method provides for unbiased estimates of wave velocity, compared to previous research. This allowed us to observe that low spatial frequency waves in the cortex propagate in the millimeter per millisecond range, the exact speed depending mainly on the temporal frequency. Previous research on the velocity of large-scale TWs in cortex has analyzed one [14,27,28,35,37], or at most a few [30,36] frequency bands. We analyzed velocity over the range 0.5 to 28 Hz. Observed velocities are closely tied to temporal frequency: the higher the temporal frequency, the higher the velocity. Earlier findings are entirely consistent with this relationship, discounting for the range of measurement and numerical methodologies. Velocities of 3–4 m/s are found for slow waves [14], 4 for theta [36] and 4–8 for alpha [27,28,30,36].

The results of our own research can be summarized by the speed (|*v*|) relationship

|v|=fλ (17)

Since the modal wavelength, *λ*, was 33 cm, and the mean normalized velocity over all samples was 0.36, the modal speed in the present results, over the temporal frequency (*f*) range 0.5 to 28 Hz, was 0.06 to 3.6 m/s. This estimate is the speed of the wave in the coordinates of the measurement array, along the diameter defined by the trajectory of the wave model. A number of scaling factors need to be applied to get from this velocity estimate to the velocity estimate at the surface of the cortex. The first of these is the conversion from the wavelength along the diameter of the MEG array to the inner surface of the array (×~π), followed by the reduction of the radius of MEG array surface to the radius of the cortical surface (÷~1.3) [Omega 2000 Whole-Cortex MEG System User Guide]. Together these scaling factors bring the speed measurements approximately in line with the measurements made in the EEG by other researchers (i.e. 0.14 to 8.6 m/s over our reported temporal frequency range). However, the incorporation of a standing wave component in the normalized speed lowers our own estimates of wave speed compared to the previous reports (×~0.38 for samples with strong wave activity). If we convert the present data from the mean normalized speed to mean speed at the scalp for the delta (2 Hz), theta (7 Hz), alpha (10 Hz) and beta (25Hz) bands, we get 0.59, 1.9, 2.6 and 6.0 m/s, respectively. Lastly, the estimate of the cortical folding factor (×~2.2) can be incorporated [28]. However, we find it appropriate to remain in the measurement coordinates used in the wave model, i.e. the Cartesian coordinates of the MEG array. This is because the filtering of velocities by the scalp and other tissues has a more profound effect on mean velocity estimates than the cortical folding factor, as we shall outline. We are content to leave the velocity estimates in the coordinate system on the outside of this low-pass filter.

### Meaning of the observed velocity values

In the TW literature, velocities are generally related to specific fiber systems of the cortex. For example, slow waves are presumed to propagate through grey matter connections [14], while alpha band phase velocities are commensurate with the conduction velocities of white-matter tracts [17,28,36]. Phase velocity is then explained as a direct function of the propagation delays introduced by the specific fiber system [49,59,60]. Here we offer a complementary explanation for the observed distribution of phase velocities, which is not inconsistent with fiber conduction velocities, but shows that they do not offer a sufficient explanation. The two addendums in this explanation are a range of *group velocities* of the cortical signal and the extra-cortical tissue acting as a *low pass filter*.

First, while phase velocity is proportional to temporal frequency, we measure the group velocity of the wave. The group velocity is not necessarily an indicator of the temporal frequency [32]. Perturbation of cortical activity creates propagating signals at a range of group velocities, and in a nonlinear relationship, a range of spatial frequencies. In the ECoG, for example, spatial spectra show no dependence on temporal frequency [40]. The wide range of velocities is supported by the entire gamut of axonal fiber types. At one end of the velocity spectrum are the slow conducting, small diameter axons within the grey matter [14]. At the other end of the velocity spectrum, there are very long-range white-matter axons, firing distal synapses in very small numbers. These fast propagating signals can flip the cortical state from one attractor to another [11]. This is possible because the cortex acts as a poised system, requiring little energy to transition from one basin to another [61].

Second, it is well understood that the skull and other intervening tissues distort the cortical signal, causing it to be blurred when measured outside the scalp. This blurring is in effect a low pass spatial and temporal filter [1], which arises through smoothing local differences in extracellular dendritic potentials [40]. Specifically, the extra-cortical tissue removes most of the signal with a spatial frequency higher than 0.1 c/cm.

Considering spatial filtering in combination with the grouping of phase velocities into packets, it can be seen that a broad range of group velocities are detectable at each temporal frequency, and these are filtered by the skull and other tissues such that the long wavelength components dominate the measured signal. Relating this to Eq 17, the temporal frequency (*f*) has detectable power across a broad range in the MEG signal, but the wavelength (*λ*) is strongly determined by the low-pass filter of the extra-cortical tissues. In addition, it has been suggested [32] that the (quasi-spherical) boundary conditions of global cortex create, via destructive interference, standing waves of the general sort that we describe in Eq 14 i.e. global waves of which the normalized velocity is between zero and unity.

Together these observations account for the repeated findings of ~30cm waves in EEG and MEG literature, across a wide range of temporal frequencies: slow wave [14], delta [15], theta [36], alpha [17,45], beta [1], gamma [35]. It also accounts for the fairly tight linear relationship between temporal frequency and wave speed in the present results, such that |*v*| is proportional to *f*. In short, measured wave speed increases with temporal frequency because of approximately constant spatial filter characteristics, not cortical conduction velocities, *per se*.

We conclude that phase velocities measured at the scalp cannot be related in a simple, direct manner to specific fiber systems, even though specific fiber systems will dominate the measured signal at specific frequencies e.g. cortico-cortical fibers for alpha band TWs in EEG [17,28]. It is difficult to detect delta-band activity propagated by fast myelinated fibers, or alpha-band activity propagated by slow grey-matter fiber, simply because the corresponding spatial wavelengths are filtered by the extra-cortical tissues or other measurement characteristics. To account for the measured relationship we need to posit a wide range of group velocities acting in concert with a low-pass spatial frequency filter, rather than add up the synaptic delays, as is common currency. Similar conclusions have recently been reached in relation to TWs in the human hippocampus [62].

Different measurement methods i.e. EEG and ECoG will yield spatial frequency spectra different from MEG, depending upon the exact spatial filter characteristics of the measurement. Simultaneous measurement methods may be required to characterize the full spatial spectrum, given that ECoG arrays can only cover a fraction of the cortex and use of small-scale arrays are in effect a high-pass spatial filter, and the MEG and EEG measure signals are low-pass filtered by the scalp. Interestingly, measurements of local field potentials (array < 1cm^2^) in the gamma range reveal that phase velocity increases also linearly with temporal frequency between 20 and 80 Hz [11], consistent with the above arguments. Notably the velocity range in this ECoG data is different to the MEG, EEG and 10cm scale ECoG, consistent with the argument that there is relatively little overlap between the two kinds of signal measurements [32].

To a first approximation, we may expect the magnitude of phase velocity measured in the MEG to be linear with temporal frequency over a wide range of brain states. However, this is mainly due to the spatial frequency filter characteristics of the intervening tissues. At the scalp, we are really only measuring one quantity: this is the velocity/temporal-frequency at 30cm wavelengths. Note that this argument can be turned on its head. Many previous findings about temporal frequency (e.g. alpha idling) can be interpreted as findings regarding scalp velocities in the range 3.0 m/s. We suggest that changes in temporal frequency measured at the scalp are largely synonymous with changes in wave speed. As power at different temporal frequencies changes according to task demands, so does average wave velocity at this specific, long wavelength.

Potential sources of error in the measurement of TW velocity include modulation in the frequency during a wave [11]. Some waves have the appearance of a spatio-temporal chirp (D. Alexander, unpublished observation), and improving the model fit to this class of waves would require modulation of the model temporal frequency during the course of the wave. The present methods can also only detect the group velocity of the wave; whether and when the measured waves are the result of more than one overlapping wave event is unknown. Such may be the case when standing waves occur, for example. Spiral waves are also often reported [10,16]; the present method only detects linearly propagating waves. Given these sources of error, the present techniques are likely to be an underestimate of the number of globally coherent wave episodes.

### Implications for static, localized sources and modular networks

Source localization is a common procedure in neuroscience, along with the decomposition of activity into localized, modular networks of activity. Freeman and Barrie [11] list three reasons why radial (bulls-eye) TWs cannot be attributed to local sources or modules; the same arguments apply to the linearly propagating, large-scale TWs described in the present research.

First, the wave may be attributed to purely physical characteristics of the spreading electro-magnetic field from a local source. However, the observed phase modulation is too large to be accounted for by the reactive component of the cortical impedance vector [63], it must therefore reflect neural activity rather than biophysical response of the grey matter [64].

Second, the wave may be attributed to a set of noise generators whose activity decreases in correlation with distance. However, this does not account for the specific spatio-temporal pattern comprised by TWs. It may be that a large number of sources activated in sequence give the appearance of a TW, but at this point the objection becomes moot. We still want to understand the functional significance of the spatio-temporal sequence of local activations that is organized very much like a TW. Similarly, a set of noise generators smeared by the skull and other intervening tissues cannot account for the observed patterns. Specifically, such blurring effects operate as low-pass spatial and temporal filters [1,38]; they cannot account for coherent motion in the signal with its symmetry in space and time.

Third, the observed TW may be attributed to the entrainment of coupled oscillators. However, the phase gradient in a TW persists over time so it would have to be considered as a phase-lagged system of oscillators, with a phase gradient mimicking the pattern of an apparent TW. See the second objection.

Several other potential objections have been dealt with previously [7]. Because similar patterns occur in both the MEG and EEG, they cannot be attributed to referencing effects which only apply to the EEG. Because similar patterns occur in the ECoG [7,11,13,65], they cannot simply be attributed to the low spatial resolution of the MEG sensor array.

Not only cannot TW activity be attributed to localized sources or networks of semi-independent modules, the former is often mistaken for the latter. According to Nunez and Srinivasan, 2006, activity measured at the surface of the scalp is a combination of TWs, standing waves and localized activity. We have previously analyzed the contribution of phase, TWs and site-wise power to the magnitude of event-related activity. We showed that the amplitude of trial-averaged activity—in the MEG, EEG and ECoG—was primarily a function of summation of phase over trials, rather than modulation of single-trial power, *per se* [7]. Further, trial-wise summation of TW models reproduces the spatial pattern of localized ‘sources’ found in trial-averaged raw time-series (ERs), and in trial-averaged maps of site-wise power. These previous results show that site-wise power, along with the cross-trial interference pattern of summed phases and summed TWs, each contribute to the localized, static modules observed in the ER.

We can further clarify what is being measured in the ER. First, the cortex exhibits activity with a broad range of spatial (and temporal) frequencies, due to different phase velocities propagated through the family of cortical fiber types, and their combination into group velocities measurable as electromagnetic waves [1,32]. Second, measurements are made outside the scalp using the EEG or MEG, resulting in detection of low-pass spatial components of the signal, while high spatial frequency components are diminished or lost. TWs in these low pass spatial components have an average normalized velocity of 0.4, as we have shown here. These TWs explain more than 50% of the variance in the phase topography measurable at the scalp (Alexander et al., 2006; 2013). Third, signals are averaged over trials, removing much of the information about the coherent motion; as also shown by the present results. *K*-means clustering indicated a diversity of spatially coherent dynamical regimes which is lost when trial-averaging is performed. This is because the procedure of trial-averaging creates an artificial interference pattern that washes out non-additive portions of the phase. Information about site-wise power that is consistent across trials is preserved, since it is a scalar quantity with appropriate statistical properties (often with a single mode, for example, albeit with a lot of trial-to-trial variation). Similarly, consistent peaks and troughs in the cosine of the phase are preserved in the trial-average, here because phase is a complex-valued quantity being averaged as if it were a scalar. Information about phase outside the peaks and troughs is lost, along with information about phases that are dispersed from the mean phase. This second component, the phase-consistency component, dominates the ER signal, relative to the power topography (Alexander et al., 2013), even while the two components provide similar information.

Let us consider the meaning of applying source localization techniques at this point. Even where the results of source localization are well-related to behavior, this relation is obtained in a stochastic sense only. The dominant contributor to the ER is the trial-averaged phase, and we will focus on this component. Source localization techniques are essentially filters applied to remove long-range correlations in the signal [54]. These filters are similar in operation to edge-detection kernels in image processing. They operate under the assumption that the rate of change in the amplitude of the trial-averaged ER across the scalp reveals the source of the signal—in this context the ER topography is treated as indicator of current source density (or the magnetic field equivalent). But since the ER is dominated by phase consistency rather than voltage amplitude (or magnetic field intensity), *per se*, the filter is locating the maximum in the cross-trial interference pattern of phase peaks. The specific location of the most consistent peak in phase is a useful quantity—as shown by the many source localization results consistent with other functional imaging. These consistently related peak locations, as part of coherent activity moving in real time, can support adaptive flexibility of behavior. But sources are more complicated and indirect entities that the world ‘source’ actually implies.

Based on the current findings, combined with previous ones in MEG and EEG (see introduction), we conclude that coherent waves on the MEG array have generically measurable speeds. Estimates of the velocity of the waves show a broad distribution of propagation speed, from standing waves to TWs. The distribution of estimated speeds is robust to event selection criteria; wave events were chosen from either (a) a particular latency, (b) as the best fit within the trial, or (c) as a sub-average of similar trials. The cortical fields—including maxima and minima in field strength—are moving over millimeters in milliseconds. The general characteristic is that sources are not static; they move. This was shown in the present research by analyzing the velocities at the characteristic latencies and frequencies of the motor and sensory evoked activity. While the direction of waves was perturbed from the average at these regions of interest, the speed of the waves was not slower; as might have been expected for the case of localized, static, sources.

We observed that there was a bimodal distribution in MEG waves, along the left-right axis. In the EEG, there is also a bimodal distribution of waves, but along the anterior-posterior axis [33]. This difference between the present MEG results and previous EEG suggests that the bimodal distributions are not the result of (simply) the left-right symmetry of the cortex or the left-right symmetry in the pattern of thickness of the skull. We hypothesize that this difference in the bimodal axis is related to the right-hand rule, associating the electric and magnetic fields of the brain. Rather than being solely a phenomenon at the local level of cortex—of static, localized magnetic fields and their equivalent current dipoles—we suggest that the physics of electromagnetism is also consistent at the global scale of interest here in the motion of the waves themselves. A test of this hypothesis would be to show that linearly propagating EEG waves are associated with spiral MEG waves whose normal vector is aligned with the trajectory of the EEG waves, and in the case of EEG spiral waves, the obverse. In this fashion, globally coherent waves can co-exist with local abeyance of the right-hand rule. Such waves will sum as an interference pattern over trials [7], thereby creating the characteristic pattern of a localized, mean-field magnetic source and equivalent current dipole.

This observation clarifies the relationship between dynamical approaches to brain function and approaches that emphasize localization of *static* sources. The sources arise from moving fields that arrive ‘just in time’ for activation of task-relevant brain regions. When considered as a cross-trial average, the resultant interference pattern has maximum amplitude over the localized brain region that is critical for the execution of the task. Of great interest is the role of the moving fields before and after they reach this localized brain region.

The present research is the first time that the velocity of large-scale cortical traveling waves have been measured over a wide range of temporal frequencies at the single trial level, using techniques that can capture both the trajectory and the speed of the waves. We found that speed scales linearly with temporal frequency. Waves that are measured outside the scalp are low-pass filtered by the intervening tissues. In combination with a range of group velocities in the cortical medium, this means that such measurements are weak indicators of physiological properties such as fiber conduction velocities, but good indicators of processes involved in global coordination of cortical activity.

## Supporting Information

S1 Fig Single trial comparison of methods. A single trial is analyzed using either the phase-unwrapping procedure and scalar regression (upper row), or complex-valued phase and multi-grid search (middle row). The trajectory and speed components show the same qualitative pattern, not dependent on the method used. Fits greater than 0.7 are shown within the white boundaries, for the case of multi-grid search but the boundaries were almost identical for both methods. The bottom row of the figure shows the expected ranges of values due to fitting procedure. Phases were randomized, and the same procedures as the middle row of panels otherwise followed.
(PDF)

S2 Fig Distribution of peak spatial frequencies as a function of size of the measurement array. The plot shows that the long wavelength (~30cm) peak is spatial frequency spectra is stable for measurement arrays of 10cm or larger i.e. scale three or bigger. Only the smallest measurement scale (~4cm array) has a different peak, with a wavelength of 10cm. Measurement scale zero corresponds to an array size of up to 7 sites (one site plus nearest neighbours) or ~4cm. Scale one corresponds to an array size of scale zero plus nearest neighbor sites. Subsequent scales are defined iteratively according to this schema. Scale ten includes almost the entire array.
(PDF)

S1 Movie Example traveling wave and quantification of its properties. A travelling wave moves in the superior to inferior direction. Its dominant direction of flow over the scalp is quantified and shown as an arrow. The wave has a temporal frequency of 7Hz, and its behavior is shown over a time-window centered on 176ms, the time of the visual dipole. Left and right views of the head are shown on the left and right, respectively. Units are cosine of phase, with hot colors positive and cold colors negative. The various wave quantities are represented by an arrow, though this is simply shorthand for the overall flow of phase over the surface of the scalp. The velocity of the wave is proportional to the distance between the center of the head and the base of the arrow (traveling waves furthest out, standing waves closest in). The wavelength of the wave is proportional to the length of the arrow (the mean wavelength of the waves is approximately the same as the size of the head, and there is not much variation in wavelength). The direction of the wave, that is the overall direction of flow, is given by the direction of the arrow, and is also colour coded so that the anterior-posterior axis is green, the inferior-superior axis is blue and the left-right axis is red. Combinations of these cardinal axes are shown as weightings of these three colours. The fit to the wave model is shown by transparency of the arrow (poor fits are transparent).
(MPG)

S2 Movie The overall distribution in the direction of cortical waves. Ongoing wave activity shows a broad range of possible directions, with a bimodal distribution along the left-right dimension. These data are for all subjects and all conditions, over all trials (arrows) and all times within a trial (movie frames). The wave model for 1.3Hz signal is shown. Conventions are the same as S1 Movie.
(MPG)

S3 Movie Task-dependent biases in the direction of cortical waves. At 176ms, 7Hz, there is a preponderance of left to right, superior to inferior traveling waves. At other times the distribution is more uniform. The movie shows the model wave vectors for a single subject (O9), condition predictable right, across all trials (arrows) and changing over the course of the trial (movie frames). Conventions are the same as S1 Movie.
(MPG)
