# Supplementary material for: Global Neuromagnetic Cortical Fields Have Non-Zero Velocity
Source: PLoS One. 2016 Mar 8;11(3):e0148413. doi: 10.1371/journal.pone.0148413 (PMC4783027; doi:10.1371/journal.pone.0148413)

Linear regression of unwrapped phases vs.
multi-grid search of complex-valued phase
Comparison to randomized phases

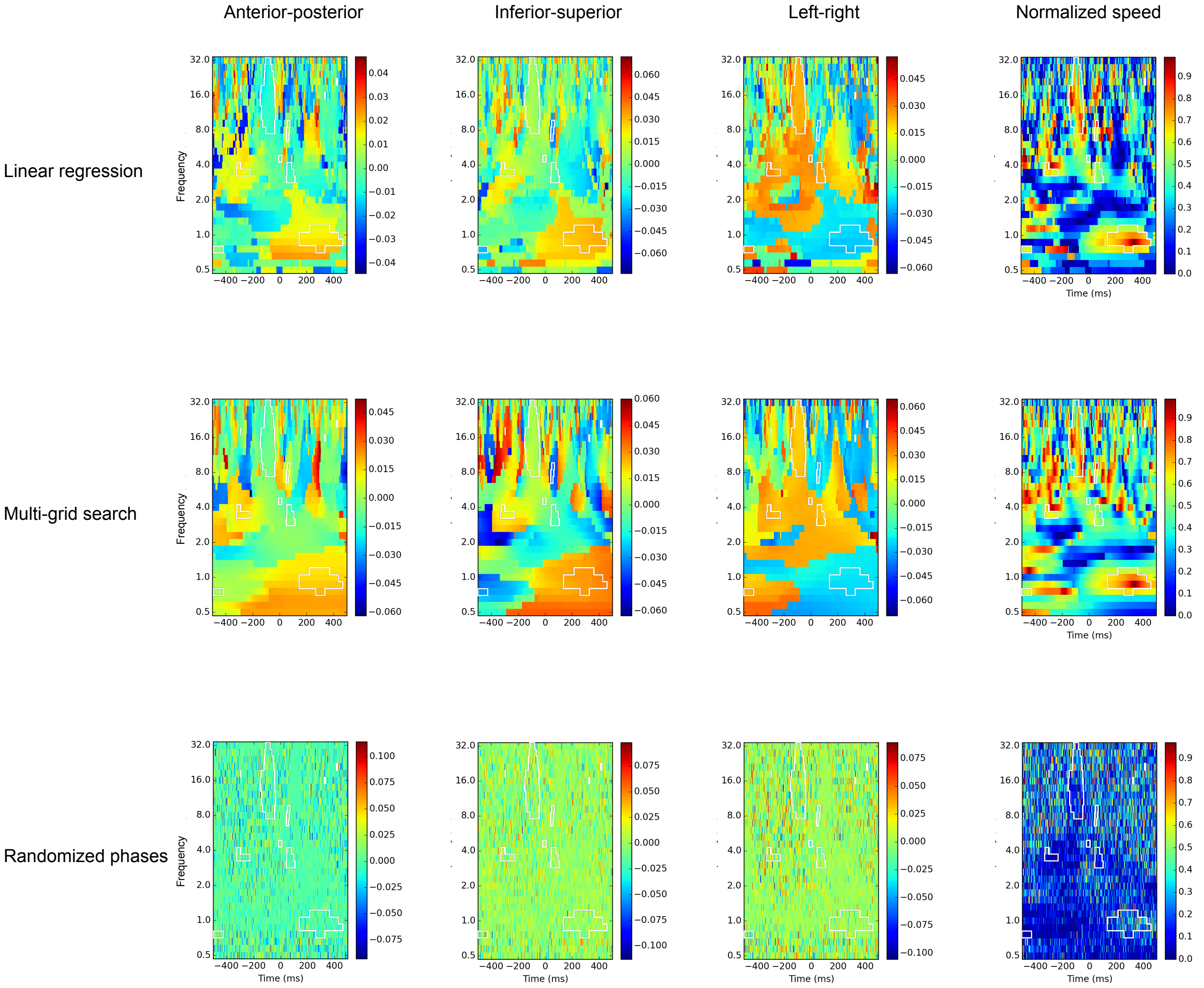

Supplement: S1 Fig — A single trial is analyzed using either the phase-unwrapping procedure and scalar regression (upper row), or complex-valued phase and multi-grid search (middle row). The trajectory and speed components show the same qualitative pattern, not dependent on the method used. Fits greater than 0.7 are shown within the white boundaries, for the case of multi-grid search but the boundaries were almost identical for both methods. The bottom row of the figure shows the expected ranges of values due to fitting procedure. Phases were randomized, and the same procedures as the middle row of panels otherwise followed. (PDF) [file pone.0148413.s001.pdf]
