# Supplementary figures and images for: Global Neuromagnetic Cortical Fields Have Non-Zero Velocity
Source: PLoS One. 2016 Mar 8;11(3):e0148413. doi: 10.1371/journal.pone.0148413 (PMC4783027; doi:10.1371/journal.pone.0148413)

## Wavelength at peak spatial frequency

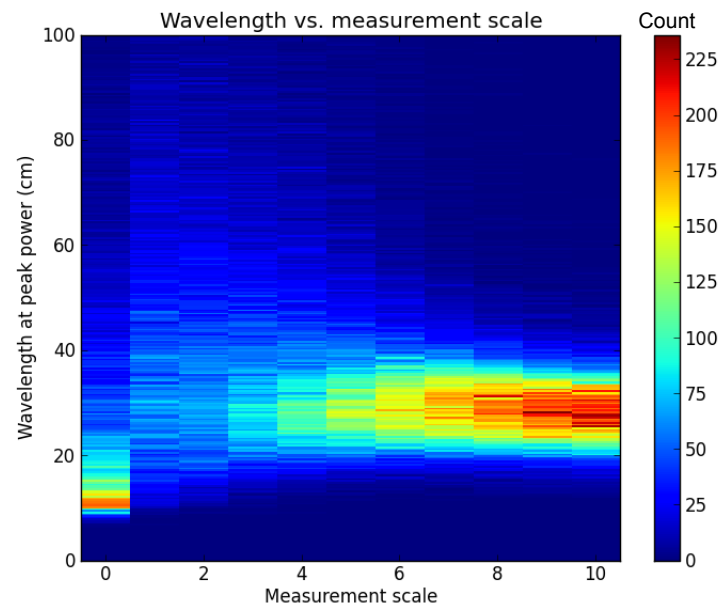

Supplement: S2 Fig — The plot shows that the long wavelength (~30cm) peak is spatial frequency spectra is stable for measurement arrays of 10cm or larger i.e. scale three or bigger. Only the smallest measurement scale (~4cm array) has a different peak, with a wavelength of 10cm. Measurement scale zero corresponds to an array size of up to 7 sites (one site plus nearest neighbours) or ~4cm. Scale one corresponds to an array size of scale zero plus nearest neighbor sites. Subsequent scales are defined iteratively according to this schema. Scale ten includes almost the entire array. (PDF) [file pone.0148413.s002.pdf]
